# Quantification of Unmethylated Alu (QUAlu): a tool to assess global hypomethylation in routine clinical samples

**DOI:** 10.18632/oncotarget.7233

**Published:** 2016-02-07

**Authors:** Raquel Buj, Izaskun Mallona, Anna Díez-Villanueva, Víctor Barrera, Dídac Mauricio, Manel Puig-Domingo, Jordi L. Reverter, Xavier Matias-Guiu, Daniel Azuara, Jose L. Ramírez, Sergio Alonso, Rafael Rosell, Gabriel Capellà, Manuel Perucho, Mercedes Robledo, Miguel A. Peinado, Mireia Jordà

**Affiliations:** ^1^ Institute of Predictive and Personalized Medicine of Cancer (IMPPC), Badalona, Barcelona, Spain; ^2^ Germans Trias i Pujol Health Sciences Research Institute (IGTP), Badalona, Barcelona, Spain; ^3^ Department of Endocrinology and Nutrition, University Hospital Germans Trias i Pujol, Badalona, Barcelona, Spain; ^4^ ISCIII Center for Biomedical Research on Diabetes and Metabolic Associated Diseases (CIBERDEM), Madrid, Spain; ^5^ Department of Pathology and Molecular Genetics, University Hospital Arnau de Vilanova and University of Lleida, Biomedical Research Institute of Lleida (IRBLLEIDA), Lleida, Spain; ^6^ Catalan Institute of Oncology (ICO-IDIBELL), L'Hospitalet de Llobregat, Barcelona, Spain; ^7^ Catalan Institute of Oncology (ICO), Hospital Germans Trias i Pujol, Badalona, Barcelona, Spain; ^8^ Catalan Institution for Research and Advanced Studies (ICREA), Barcelona, Spain; ^9^ Hereditary Endocrine Cancer Group, Spanish National Cancer Research Center (CNIO), Madrid, Spain; ^10^ ISCIII Center for Biomedical Research on Rare Diseases (CIBERER), Madrid, Spain

**Keywords:** DNA hypomethylation, Alu repeats, human cancer, biomarker, routine clinical biospecimens

## Abstract

Hypomethylation of DNA is a hallmark of cancer and its analysis as tumor biomarker has been proposed, but its determination in clinical settings is hampered by lack of standardized methodologies. Here, we present QUAlu (Quantification of Unmethylated Alu), a new technique to estimate the Percentage of UnMethylated Alu (PUMA) as a surrogate for global hypomethylation.

QUAlu consists in the measurement by qPCR of Alu repeats after digestion of genomic DNA with isoschizomers with differential sensitivity to DNA methylation. QUAlu performance has been evaluated for reproducibility, trueness and specificity, and validated by deep sequencing. As a proof of use, QUAlu has been applied to a broad variety of pathological examination specimens covering five cancer types.

Major findings of the preliminary application of QUAlu to clinical samples include: (1) all normal tissues displayed similar PUMA; (2) tumors showed variable PUMA with the highest levels in lung and colon and the lowest in thyroid cancer; (3) stools from colon cancer patients presented higher PUMA than those from control individuals; (4) lung squamous cell carcinomas showed higher PUMA than lung adenocarcinomas, and an increasing hypomethylation trend associated with smoking habits.

In conclusion, QUAlu is a simple and robust method to determine Alu hypomethylation in human biospecimens and may be easily implemented in research and clinical settings.

## INTRODUCTION

Extensive evidence describes cancer as a combination of genetic and epigenetic alterations which cooperate at every step of the tumor progression (reviewed in [1]). DNA methylation is the most well-characterized epigenetic mark in mammals and consists in the covalent addition of a methyl group to the cytosine located within the CpG dinucleotide. It is frequently associated with silenced chromatin and transcriptional repression (reviewed in [2-3]). Among all the epigenetic alterations that delineate cancer genomes, loss of global DNA methylation has been considered a hallmark. Numerous works have demonstrated that DNA hypomethylation is an early and sustained event in tumorigenesis. Besides, it promotes a permissive landscape for cancer development and progression by encouraging chromosomal instability, imprinting loss, aberrant gene expression and transposon activation (reviewed in [2-3]). More importantly, it has been reported a strong association between the degree of DNA hypomethylation and the tumor grade and stage, which has attracted great interest for its potential clinical value, not only in cancer diagnosis and prognosis [4-9], but also as a marker of cancer risk [9-12].

A wide variety of techniques have been designed to measure global DNA methylation, some of which quantify the overall levels of 5-methylcytosine in the genome compared with unmethylated cytosines (e.g. HPLC, immunochemical assay, etc.), while others assess the methylation levels of specific genome compartments (reviewed in [9, 13]). Among the second group, the most widely used methods are those based on repeat elements, as they exhibit a high copy number and are widespread throughout the human genome. Nevertheless, none of them has been established in the clinical practice due in part to technical, economical and time shortcomings, which preclude a standardized alternative.

Here we present a new method, Quantification of Unmethylated Alu (QUAlu), which uses Alu repeats as surrogate reporter of global DNA methylation. Alu repeats are primate-specific transposable elements that belong to the Short Interspersed Elements (SINEs) family and represent the most abundant class of repetitive sequences in the human genome (1.1 million copies per haploid genome) [14]. Alu elements contain up to 25% of the overall CpG sites in the genome (Table 1) and are highly methylated in somatic tissues. Interestingly, they are located in gene-rich regions [15].

QUAlu is a simple and rapid method based on the digestion of genomic DNA with the methylation-sensitive and insensitive isoschizomers *Hpa*II/*Msp*I, the ligation of an adaptor and a qPCR using primers specific for the Alu consensus sequence. We have applied this technique to a broad variety of pathological examination samples including fresh frozen tissues, formalin-fixed paraffin-embedded (FFPE) sections, fine-needle aspiration biopsies (FNAB), stools and liquid biopsies. Our preliminary results underscore the potential clinical utility of the assessment of unmethylated Alu elements by QUAlu.

## RESULTS

### QUAlu design and technical evaluation

**Figure 1:**
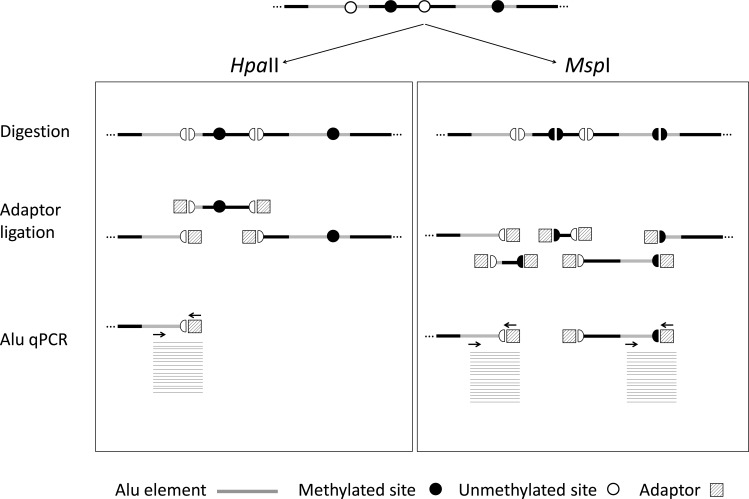
QUAlu technique diagram Genomic DNA is digested using *Hpa*II and *Msp*I isoschizomers (DNA methylation sensitive and insensitive, respectively), ligated to a synthetic adaptor, and Alu elements are specifically amplified by qPCR in two separated reactions. The ratio between the two reactions gives the percentage of unmethylated Alu elements (PUMA). DNA normalization is performed by parallel amplification of L1PA.

According to the reference human assembly hg19, there are over 28 millions of CpG dinucleotides in the human genome and more than half are located within repeat elements, being Alu elements those containing the highest fraction, namely 25.4% (Table 1). Therefore, we selected Alu elements as the most adequate surrogate reporter of global methylation and developed QUAlu technique, a method to identify unmethylated Alu repeats which shares the quantitative nature of the related technique LUMA [16] and the specificity of QUMA [17]. Fundamentals of QUAlu assay are outlined in Figure 1 and Supplementary Figure S1. QUAlu is based on the different methylation sensitivity of the isoschizomers *Hpa*II/*Msp*I (see Materials and Methods and Supplementary Material), whose recognition site is C/CGG, located in the Alu consensus sequence AACCCGG present in 14.4% of Alu elements (Table 1). Noteworthy, analysis of whole genome bisulfite sequencing data [18] showed that the *Hpa*II/*Msp*I sites embedded in CpG islands may be used as reporters of the overall methylation of these genomic elements [19]. In this regard, we also confirmed that this postulate may be also applied to Alu repeats: more than 90% of the *Hpa*II/*Msp*I sites within the Alu consensus sequence AACCCGG showed concordant methylation levels with the whole Alu sequence (Supplementary Table S4). To determine the virtual representativeness of QUAlu (QUAluome), an electronic qPCR simulation was performed showing a theoretical coverage of 155 878 Alu elements (see Supplementary Material), which corresponded to the 13.65% of the Aluome, with a bias toward amplification of young subfamilies (Supplementary Figure S2).

**Table 1:** Alu repeat content in the human genome and representativeness in the virtual QUAlu ^a^

Sequence	No of elements	Base pairs	No of CpGs	No of HpaII/MspI sites	AACCCGG hits^d^	QUAluome^d^
Alu ^b^	1,194,734	305,076,148 (9.7%)	7,173,987 (25.4%)	742,725 (32.3%)	172,574 (79.1%)	155,878
LINE ^c^	1,498,690	638,481,131 (20.4%)	3,412,416 (12.1%)	155,813 (6.8%)	6,881 (3.2%)	0
CpG islands	28,691	21,842,742 (0,7%)	2,089,537 (7.4%)	270,622 (11.8%)	4,470 (2.1%)	0
Genome	-	∼3,200,000,000 (100%)	28,217,009 (100%)	2,297,221 (100%)	218,131 (100%)	0

a Data based on GRCh37/hg19 human genome assembly.

b RepeatMasker's Alu repFamily members discarding FLAMs and FRAMs.

c RepeatMasker's LINE repClass members.

d Virtual QUAlu amplicons.

**Figure 2:**
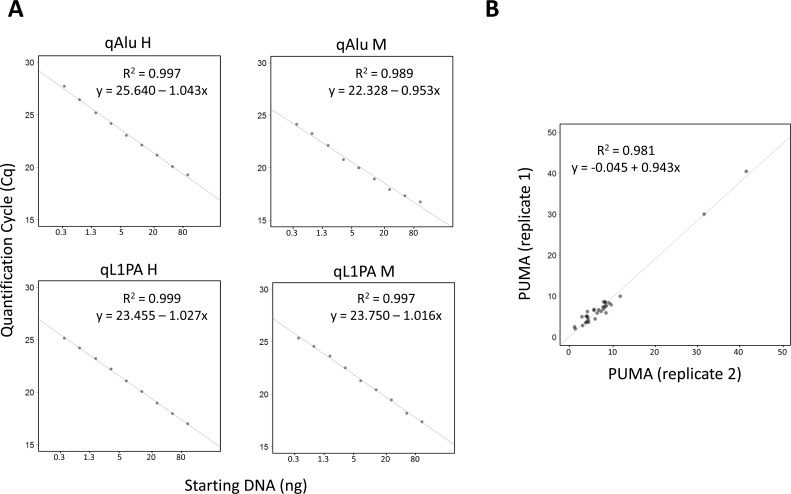
Evaluation of QUAlu technique **A**. Standard curves showing the linear range of the different qPCRs performed in a QUAlu assay. HCT116 genomic DNA amounts ranging from 0.3 to 80 ng were used. **B.** Correlation of the percentage of unmethylated Alu elements (PUMA) determined by QUAlu in two independent experiments.

To assess the linearity of QUAlu, different starting amounts of HCT116 genomic DNA, ranging from 0.3 to 80 ng, were used. As it can be observed in Figure 2A, all quantifications showed excellent linearity (R^2^> 0.98 in all cases). Moreover, similar percentages of unmethylated Alu elements were obtained, being the overall average 8.8± 2.2 (Supplementary Figure S3A). The same assay was done with clinical samples from normal tissues of lung, colon and thyroid, obtaining an excellent linearity in all cases (Supplementary Table S5).

The inter-assay repeatability was assessed by analyzing the same HCT116 genomic DNA in 39 independent QUAlu assays. The mean of all the analyses was 9.9% and the standard deviation ±1.6. Furthermore, replicates of tumor and normal clinical samples were measured in different plates confirming that the technique is reproducible (R^2^ = 0.981) (Figure 2B). Importantly, the feasibility of QUAlu in samples containing partially degraded DNA was verified (Supplementary Material and Supplementary Figure S3B-S3C).

It is of note that consistent QUAlu results may be achieved with DNA amounts well below one haploid genome. In fact, linear range response was reached with as little as 0.005 pg of DNA per PCR tube (equivalent to 0.002 haploid genomes) (Supplementary Figure S4). The low requirements of QUAlu are due to the multiplex nature of its target (a large pool of more than one hundred thousand Alu repeats), reaching sensitivity detection 4 to 5 orders of magnitude higher than a single copy locus (e.g. promoter region of Digestive organ expansion factor homolog (zebrafish) -*DIEXF*- gene) (Supplementary Figure S4 and Supplementary Material). Noteworthy, the complexity of the QUAlu product is visualized as a broad melting peak for both the *HpaII* and the *MspI* samples, contrasting with the narrower peak of a single PCR product (Supplementary Figure S5).

Finally, the specificity of QUAlu to amplify Alu elements was validated by next-generation sequencing of five QUAlu determinations. The results showed that 97% of the reads (range 96.6-98.1%) obtained from the sequenced samples aligned with Alu repeats (Supplementary Table S6) and confirmed the complex composition of QUAlu product composed of multiple different Alu elements with similar distributions among all the analyzed samples (Supplementary Figure S6).

### QUAlu application to fresh frozen human cancer samples

**Figure 3:**
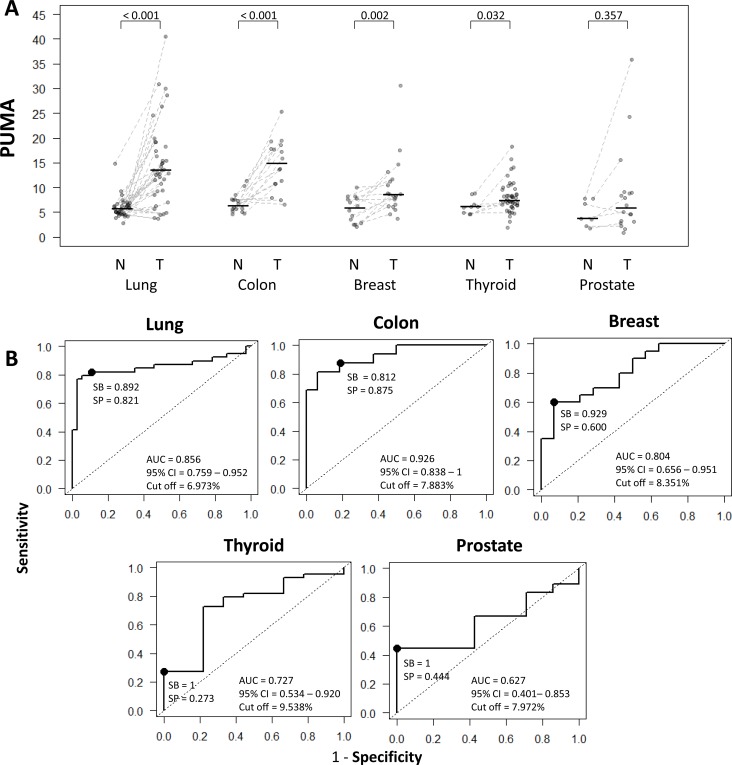
Comparison and diagnostic value of QUAlu among different cancer types **A**. PUMA in different cancer types (normal tissue (N) and tumor (T)); the median of each group is represented by a black line. **B**. Receiver Operating Characteristic curves for the diagnosis of lung, colon, breast, thyroid and prostate cancer according to the percentage of unmethylated Alu (PUMA) elements determined by QUAlu.

QUAlu technique was applied to analyze the levels of unmethylated Alu elements in different cancer types and their normal counterparts (Table 2 and Supplementary Table S7). Interestingly, the different normal tissue types showed similar values of Percentage of UnMethylated Alu elements (PUMA) (Table 2) (Kruskal Wallis test, *p*-value = 0.308), being the average PUMA 6.0 ± 2.1 (range 1.8-14.8). However, PUMA showed a broad variation in tumors, ranging from 0.9 to 40.5 (average = 10.7 ± 6.8). Furthermore, important differences were observed between cancer types (Table 2 and Figure 3A) (Kruskal Wallis test, *p*-value < 0.05), with colon and lung exhibiting 2-3 fold higher levels of unmethylated Alu repeats as compared with thyroid, prostate and breast cancer (Supplementary Tables S7 and S8).

**Table 2:** Percentage of Unmethylated Alu repeats (PUMA) in different human tissues and tumors

	Thyroid	Prostate	Breast	Colon	Lung
Normal tissue	6.2 ± 1.6 (n = 9)	4.8 ± 2.6 (n=7)	5.6 ± 2.66 (n=14)	6.9 ± 1.9 (n=16)	5.9 ± 2.0 (n=37)
Tumor tissue	8.2 ± 3.1 (n=59)	8.5 ± 8.8 (n=18)	10.0 ± 5.9 (n=20)	14.6 ± 5.2 (n=16)	14.6 ± 8.3 (n=39)
p-value ^a^	0.032	0.357	0.002	< 0.001	< 0.001

a Normal vs. Tumor, Mann-Whitney U test

The comparison between normal and tumor samples showed statistically significant differences in most cancer types, except in prostate cancer (Mann-Whitney U test, *p*-value = 0.357) (Figure 3A and Table 2).

Since there were no differences among normal tissues in the proportion of unmethylated Alu elements, the 99^th^ percentile (PUMA = 12%) was taken as a cut-off value to consider a tumor as hypomethylated. Thus, particular analysis of each tumor type revealed that 62.5% of colon and 64.1% of lung tumors were hypomethylated, while in breast and prostate tumors this figure was 20% and 16.7%, respectively. Otherwise, only 11.9% of thyroid tumors had percentages of unmethylated Alu elements above the reference value (Supplementary Figure S7). When considering only the matched normal-tumor pairs of all cancer types (*n* = 81), the difference among them was evident in most cases (paired Mann-Whitney U test *p*-value<0.001). About one third of colon (5/16) and lung (13/37) tumors displayed a high hypomethylation as compared with the paired normal tissue (fold change greater than 3). Contrarily, this big difference was uncommon in breast (2/14), prostate (1/7) or thyroid (0/7) cancer (Supplementary Tables S7 and S9).

### Evaluation of PUMA as biomarker in specific cancers

To estimate the potential value of the percentage of unmethylated Alu elements to discriminate between normal and tumoral tissue we performed ROC analyses (Figure 3B). Area Under the Curve (AUC) values confirmed that PUMA was a good biomarker (AUC > 0.8) for breast, colon and lung cancer, with sensitivities and specificities >80%, except for breast cancer, whose sensitivity reached 92.9% but the specificity was lower (60%). Otherwise, for both prostate and thyroid cancer, although the sensitivity reached 100%, the specificity was low (44.4% and 27.3%, respectively). The cut-off values varied from tissue to tissue (Figure 3B), with the lowest levels in lung (6.97) and the highest in thyroid (9.54).

Moreover, to evaluate the clinical value of the QUAlu assay, we performed additional statistical analyses in two cancer types with the highest and the lowest PUMA, namely lung and thyroid cancer.

**Figure 4:**
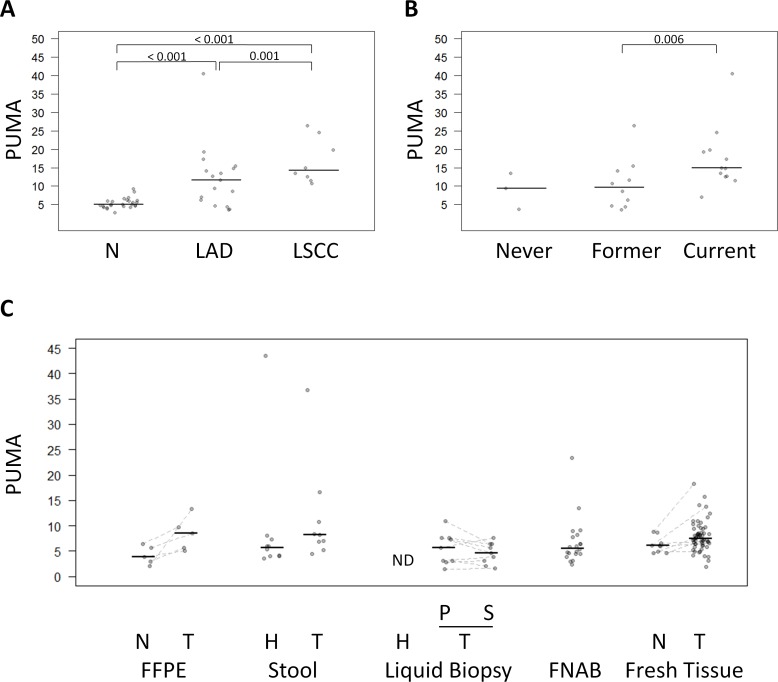
Comparison of QUAlu among different clinical characteristics and sample types **A**. PUMA in normal lung tissue, lung adenocarcinoma (LAD) and lung squamous cell carcinoma (LSCC) and **B**. in lung cancer patients according to their smoking habits. **C**. PUMA in different sample types: FFPE (colon cancer patients), stool (healthy donors and colon cancer patients), liquid biopsy (plasma from healthy donors, and plasma and serum from lung cancer patients), FNAB (thyroid goiter patients), fresh tissue (thyroid cancer patients). The median of each group is represented by a black line. Normal (N), tumor (T), healthy donors (H), plasma (P), serum (S), no detectable samples (ND).

As described above, lung cancers exhibited high levels of hypomethylated Alu elements, but differences were also detected among lung cancer subtypes. Specifically, lung squamous cell carcinoma showed the highest PUMA (16.8 ± 8.8) compared with lung adenocarcinomas (12.2 ± 6.1) (Mann-Whitney U test *p*-value = 0.001) (Figure 4A and Supplementary Table S10). On the other hand, while no significant differences were observed in PUMA among lung normal tissue from never smokers, former smokers (more than 10 years) and current smokers (Kruskal Wallis test *p*-value >0.05), there was a significant increasing trend (Mann-Whitney U test, *p*-value = 0.006) of lung tumors to become more unmethylated in current smokers compared to former smokers (Figure 4B).

As regard to thyroid cancer, there were no significant differences in the percentage of unmethylated Alu elements in relation to histology subtype (Kruskal Wallis test, *p*-value = 0.231) or genetic alteration (RAS or BRAF mutations) (Kruskal Wallis test *p*-value = 0.147).

### QUAlu application to diverse pathological examination biospecimens

Next, we evaluated the applicability of QUAlu to biospecimens obtained in standard pathological procedures often containing low amount of poor quality DNA (FFPE, FNAB, liquid biopsies and stools). Due to the low amount of starting material, DNA was not quantified and 1 ul of the extracted DNA was used for QUAlu analysis (see Supplementary Material). All samples produced detectable levels of amplified Alu elements (qAlu M Cq range: 16 to 30, qAlu H Cq range: 19 to 34) (Supplementary Figure S8), with the exception of plasma samples obtained from healthy individuals and one stool sample from one colon cancer patient.

The low number of samples precluded a robust comparison of the results, but some insightful trends were observed (Figure 4C). FFPE colon tumors showed higher PUMA than the matching normal samples (paired Mann-Whitney U test *p*-value = 0.062). Moreover, elevated Alu hypomethylation in the stools was more frequent in colon cancer patients than in control individuals (Mann-Whitney U test *p*-value = 0.079). Interestingly, liquid biopsies produced similar PUMA in plasma and serum from lung cancer patients, but did not amplify in cancer-free controls, which is consistent with the common absence of circulating free DNA in healthy individuals [20].

Finally thyroid goiters FNABs showed a PUMA in the same range than normal tissues with some exceptions: four cases presented a PUMA above 8.8, the highest value in normal thyroid tissue (Figure 4C).

## DISCUSSION

Most of human genome is methylated, but in a wide range of pathologies, including cancer, a global DNA hypomethylation occurs affecting in large extent repetitive elements, which constitute ∼45% of the genome. Weisenberger et al. [21] demonstrated that the methylation of different repetitive sequences, namely, LINE-1, Alu and satellite 2 (Sat2), significantly correlated with global methylation levels measured by high performance liquid chromatography (HPLC), and proposed the use of these repeats as surrogate reporters of global methylation. LINEs have been broadly used to estimate the global levels of hypomethylation [22-24], but Alu elements display some features that make them more suited for this purpose. Namely, Alu repeats constitute the most abundant retrotransposon and contain 25% of all the CpGs in the human genome. Moreover, due to their prevalent localization in gene-rich regions [15] epigenetic variations in Alu repeats may have direct implications in gene regulation, and by extension, in tumor biology.

Here we present QUAlu, a new technique to measure the levels of DNA unmethylation in Alu repeats, with several features that facilitate a direct implementation in clinical and research settings. These include a 100 fold higher sensitivity than other related methods [25], as accurate determinations can be performed with as little as 300 pg of DNA (corresponding to approximately 150 diploid cells). QUAlu specificity for Alu elements is extremely high, as demonstrated by deep sequencing of its products, where 97% of the reads mapped in Alu repeats.

Moreover, thanks to the calibration with internal controls (L1PA simultaneously with Alu repeats), QUAlu is relatively unaffected by the quantity and quality of the starting material in artificially degraded DNA. Furthermore, we have demonstrated that this technique is amenable to be applied to a broad spectrum of pathological examination specimens routinely collected in clinical settings (frozen tissues, FFPE, liquid biopsies, stools and FNAB). In spite of the low number of samples analyzed, preliminary results are promising, especially for FFPE and stools. Nevertheless, direct applicability of QUAlu in different clinical settings requires the analysis of large series of cases to define the threshold, sensitivities and specificities.

With clinical practice in mind, technical benefits of QUAlu include the small number of steps (digestion-ligation, real time PCR and analysis), the short time required to complete the determination (less than 5 hours from the DNA to the final result, even without automation of the process), and the low cost (about 6.3 US$ per sample, including technician labor).

As mentioned above, several techniques have been developed to estimate global methylation, and many of them target repeat elements as surrogate reporters (reviewed in [9, 13]). While most of these methods may constitute good alternatives to compare global methylation levels among a few samples, their implementation as a clinical tool is not a straightforward approach due to either technical complexity or exquisite sample necessities. QUAlu simplicity and limited equipment requirements facilitate its implementation in most laboratories.

To assess the clinical potential of QUAlu, we determined the extent of Alu hypomethylation in different human cancers by analyzing normal and tumor tissues. In a first analysis we compared the different normal tissues and different individuals, showing that the levels of unmethylated Alu elements were very consistent from tissue to tissue and from individual to individual. This result was in agreement with previous studies analyzing global DNA methylation by MethyLight and HPLC [21] or targeting Alu elements [26-27], but not with other works reporting tissue-associated global methylation differences targeting LINE-1 [28].

Our data suggests that the degree of hypomethylation is variable among different cancer types, with thyroid, prostate and breast cancer exhibiting low levels of hypomethylation, while cancers of colon and lung displayed the highest levels. Chalitchagorn et al. [28] analyzed LINE-1 methylation using bisulfite based PCR in several cancers, and although they found high levels of hypermethylation in some types (e.g. esophagus cancer), no hypomethylation was observed among the ones analyzed in our study. This might be explained by the low sensitivity of their technique or the low number of samples analyzed.

It is interesting to note that the low-hypomethylation cancer group included hormone-related tissues (thyroid, prostate and breast) with no direct interaction with external factors, while the high-hypomethylation group (colon and lung) was composed by tumors with a high exposure to environmental factors (e.g. diet, air). While we do not know the reason for this association, there are evidences supporting the impact of certain environmental factors (drugs, chemicals, pollutants and other agents) in the deregulation of epigenetic enzymes, which will eventually generate epigenetic changes, including DNA hypomethylation, that may accumulate over the time causing alterations in key cellular processes and promoting cancer [29-30]. Noteworthy, it has been reported that Alu hypomethylation (but not LINE-1 hypomethylation) in esophageal mucosa may reflect an epigenetic field for cancerization in esophageal carcinogenesis [12]. Other alternative explanations may be related with the dynamics of tumor progression in different tumor types and the role of DNA hypomethylation behind specific deregulation of biological pathways, including genomic stability [31-32].

Confirming previous reports [33] we found significant differences in the levels of hypomethylation among the two types of lung cancers considered here. Lung adenocarcinomas, the histological subtype most frequently associated with never-smokers and former smokers, were less hypomethylated than lung squamous cell carcinomas. Many studies have suggested a strong correlation between loss of DNA methylation and smoking habit in cancer patients [34-36], but also in healthy people [37-38]. In this regard, we found a significant increase of hypomethylation in current smokers compared to former smokers, but this trend was not observed in the adjacent normal tissue. This result may indicate different mechanisms of tumor progression in ex-smokers as compared with current smokers.

In summary, we have demonstrated that QUAlu is a feasible approach to analyze global DNA methylation in almost any type of biospecimen routinely collected in ordinary clinical settings. DNA hypomethylation is a hallmark of cancer but, as we have shown, its degree is highly variable. Its determination with a technique as QUAlu may have a broad spectrum of applications including diagnostic and prognostic evaluations.

## MATERIALS AND METHODS

### Samples

This study included a total of 300 pathological examination samples of different sources: 220 fresh frozen tissue samples (Supplementary Tables S1 and S2), 10 FFPE samples, 31 liquid biopsies, 19 stool samples and 20 thyroid goiter FNAB samples. Regarding fresh frozen tissues, 16 colorectal carcinomas and their paired normal adjacent tissues were obtained from Hospital Universitari de Bellvitge (Barcelona, Spain). Forty-four thyroid carcinomas and 9 paired adjacent thyroid tissues were obtained as described in our previous study [39]. DNA of 20 breast carcinomas and 14 paired normal adjacent tissues, 18 prostate carcinomas and 7 paired normal adjacent tissues, and 39 lung carcinomas and 37 normal adjacent tissues were obtained from the Spanish National DNA Bank (BNADN, Salamanca, Spain). Patient characteristics are shown in Supplementary Tables S1 and S2. Five normal and tumoral paired colorectal carcinoma FFPE samples were obtained from Cooperative Human Tissue Network (CHTN). Nine stool samples from colorectal carcinoma patients and 10 from healthy donors were obtained from Hospital Universitari de Bellvitge (Barcelona, Spain). Finally, nine plasma and serum paired lung carcinoma liquid biopsies, 13 plasma liquid biopsies from healthy donors and 20 thyroid goiter FNAB samples were obtained from Hospital Universitari Germans Trias i Pujol (Badalona, Spain). The study was approved by the Hospital Germans Trias i Pujol Ethics Committee. Informed consent was obtained before surgery.

The colorectal carcinoma cell line HCT116 was obtained from the American Type Culture Collection (ATCC) and was authenticated on 3^rd^ March 2014 by using the AmpFLSTR^®^ Identifiler^®^ Plus PCR Amplification Kit (Applied Biosystems). Cells were cultured in D-MEM/F12, supplemented with sodium pyruvate, L-glutamine and 10% fetal bovine serum (Life Technologies, MD, USA) and were maintained at 37°C in a 5% CO_2_ atmosphere. Genomic DNA was isolated using different methods as described in the Supplementary Material.

### Human genome sequence data sets

We used the GRCh37/hg19 human genome assembly. Genomic positions of Alu elements, LINE sequences, CpG islands and CpG dinucleotides were retrieved from the UCSC MySQL repository (genome-mysql.cse.ucsc.edu). Motif search was performed with EMBOSS (http://emboss.sourceforge.net/). To count the number of overlapping features (Table 1) we used the BEDTools package (v2.19.1, [40]). For more technical details see Supplementary Material.

### Quantification of Unmethylated Alu (QUAlu) assay

The principle underlying this technique is the selective amplification of Alu repeats containing an unmethylated CpG site within the consensus sequence AACCCGG. Briefly, genomic DNA was digested in parallel in two separated tubes with *Hpa*II and *Msp*I methylation-sensitive and -insensitive isoschizomers, respectively, which leave identical sticky ends (C/CGG). Next, a synthetic adaptor was ligated to the digested DNA fragments. Quantification was performed by qPCR using a primer complementary to the chimeric sequence of the adaptor plus the consensus Alu sequence after *HpaII* digestion (AACC + synthetic adaptor) and another one complementary to the Alu consensus sequence and located ∼20 nucleotides upstream of the *HpaII* cutting site (Supplementary Figure S1). Thereby, qPCR of *Msp*I digestion (qAlu M) allowed the quantification of all the amplifiable Alu elements (irrespective of the methylation status), while qPCR of *Hpa*II digestion (qAlu H) only quantified the subset of amplifiable Alu elements containing an unmethylated CpG. Thus, the final result corresponded to the fraction of unmethylated Alu elements respect the total number of amplifiable Alu elements calculated according to the equation described below. Furthermore, two specific qPCRs for L1PA (a Long Interspersed Nuclear Element-1, LINE-1 subfamily) were performed to normalize the DNA input for both *Msp*I (qL1PA M) and *Hpa*II digestions (qL1PA H). For more technical details see Supplementary Material.

### QUAlu data analysis

Statistical analyses were performed using R version 3.1.0. The Percentage of UnMethylated Alu elements (PUMA) for each sample was assigned according to this equation:

$$\text{PUMA}=\frac{\frac{{\text{E}}_{\text{qAlu H}}{}^{{-\text{Cq}}_{\text{qAlu H}}}}{{\text{E}}_{\text{qLlPA H}}{}^{{-\text{Cq}}_{\text{qLlPA H}}}}}{\frac{{\text{E}}_{\text{qAlu M}}{}^{{-\text{Cq}}_{\text{qAlu M}}}}{{\text{E}}_{\text{qLlPA M}}{}^{{-\text{Cq}}_{\text{qLlPA M}}}}}\times 100$$

The relative amount of unmethylated Alu elements (given by qAlu H normalized by the reference sequence qL1PA (qL1PA H)) and the relative amount of total amplifiable Alu elements (given by qAlu M normalized by qL1PA M) were calculated as a ratio of exponential functions in which the base was the qPCR efficiency (E) and the variable was the quantification cycle (Cq). To tackle the qPCR error propagation, permutation tests were done to obtain a final PUMA and its variation using the qPCR R package (v1.4-0, [41]). Mann-Whitney U test and Kruskal Wallis tests were used, as appropriate, to assess the significance among the different groups of samples. Correlation analyses were conducted using two-tailed Kendall tests. The significance level was established at p<0.05 for all analyses. Receiver operating characteristic (ROC) curves were generated using the pROC R package (v1.7.3, [42]) to assess the cut-off value that best discriminated between tumor and normal tissue according to the PUMA.

### Characterization of QUAlu product by Next Generation Sequencing

To determine the specificity of the technique, the products generated by qAlu H and qAlu M from 5 samples (the colorectal cancer cell line HCT116, a lung squamous carcinoma with its normal matching tissue and a papillary thyroid carcinoma with its normal matching tissue) were sequenced using Ion Torrent technology (Life Technologies). For more technical details see Supplementary Material and Supplementary Table S3.

## SUPPLEMENTARY MATERIAL FIGURES AND TABLES




