# Supplementary material for: Quantification of Unmethylated Alu (QUAlu): a tool to assess global hypomethylation in routine clinical samples
Source: Oncotarget. 2016 Feb 7;7(9):10536–46. doi: 10.18632/oncotarget.7233 (PMC4891138; doi:10.18632/oncotarget.7233)
Supplement: Supplementary file 1 [file oncotarget-07-10536-s001.pdf]

Quantification of Unmethylated Alu (QUAlu): a tool to assess global hypomethylation in routine clinical samples

Supplementary Material

Index page

Supplementary methods

Samples 2
QUAlu protocol 2
Determination of Alu elements containing the AACCCGG motif 3
Adaptor and primers design 3
Ion Torrent library preparation, sequencing and analysis 4
Effect of DNA degradation on QUAlu performance 4
Standard curve and relative sensitivity of QUAlu versus a single copy locus 4
Tips and troubleshooting 4
List of abbreviations 5

Supplementary figures

Supplementary Figure S1 6
Supplementary Figure S2 7
Supplementary Figure S3 8
Supplementary Figure S4 9
Supplementary Figure S5 10
Supplementary Figure S6 11
Supplementary Figure S7 12
Supplementary Figure S8 13

Supplementary references 14

SUPPLEMENTARY METHODS

Samples

Primary tumors and normal adjacent tissues were snap frozen following surgery and stored at -80ºC. Genomic DNA from thyroid samples was extracted using the DNeasy Blood and Tissue kit (QUIAGEN, Valencia, CA, USA) according to the manufacturer's protocol. Genomic DNA from colon samples was isolated by standard phenol/chloroform protocol. Genomic DNA from breast, prostate and lung samples were obtained from the Spanish National DNA Bank (BNADN, Salamanca, Spain). One 10µm section of FFPE blocks were processed using the E.Z.N.A. FFPE DNA kit (Omega Bio-Tek, Norcross, GA, USA), with a xylene wash to remove paraffin and a final treatment of RNaseA (1 h at 45ºC). DNA from liquid biopsy samples was isolated with QIAamp DNA Blood Mini Kit (QIAGEN), while DNA from stools was extracted with QIAamp DNA Stool Mini Kit (QIAGEN). Genomic DNA from HCT116 cells and thyroid FNAB samples was extracted using the PureLink Genomic DNA Kit (Invitrogen, Carlsbad, CA, USA). Importantly, QUAlu assay was carried out using HCT116 genomic DNA isolated by different methods and no differences were observed in the percentage of unmethylated Alu elements (data not shown).

QUAlu protocol

For the standard QUAlu protocol, 2 aliquots consisting of 5 ng of genomic DNA (from fresh frozen tissues and cell lines) or 1uL of unknown concentration genomic DNA (FFPE, liquid biopsies, stools and FNAB samples) were simultaneously digested and ligated in two respective tubes with either 1 U of *Msp*I (International Inc., Burlington, Ontario, Canada) or 1 U of *Hpa*II (Fermentas). For the reaction 2 uL of Tango buffer 10X (Fermentas), 2 U of T4 ligase, 1 uL of T4 ligase buffer 10X (Fermentas), 1 nmol/L of synthetic adaptor (the adaptor sequence is shown below and in Supplementary Fig. S1) and water were added to a final volume of 30 uL. Mixes were incubated for 1 h at 37ºC followed by 2 h at 16ºC. Next, thermal inactivation of the enzymes was performed for 20 min at 65ºC.

	***Msp*I digestion (uL)**	***Hpa*II digestion (uL)**
**Nuclease-free water**	24.7	24.7
**10X Tango buffer**	1	1
**10X T4 ligase buffer**	2	2
**Adaptor (1 nmol/L)**	1	1
***Msp*I (10 U/uL)**	0.1	-
***Hpa*II (10 U/uL)**	-	0.1
**T4 ligase (5 Weiss U/uL)**	0.2	0.2
**DNA**	1	1
Final Volume	30	30

Real-Time PCR experiments were performed in a LightCycler® 480 Real-Time PCR System with white Multiwell Plate 384 plates (Roche Diagnostics GmbH, Mannheim, Germany). Both *Msp*I and *Hpa*II digestion-ligation mixtures were quantified using a specific Alu qPCR and a specific L1PA qPCR. A final volume of 10 uL for each reaction was prepared as follows: 5 uL of LightCycler® 480 SYBR Green I Master (Roche), 1 umol/L forward primer, 1 umol/L reverse primer (see detailed information about primers sequence below) and 1 uL of template from the digestion-ligation mixture diluted 1:20, equivalent to 0.008 ng of DNA.

	**qAlu (uL)**	**qL1PA (uL)**
**Nuclease-free water**	3.8	3.8
**Alu Fw primer (100 nmol/L)**	0.1	-
**Alu Rv primer (100 nmol/L)**	0.1	-
**L1PA Fw primer (100 nmol/L)**	-	0.1
**L1PA Rv primer (100 nmol/L)**	-	0.1
**LightCycler® 480 SYBR Green I Master**	5	5
**Digestion-ligation dilution**	1	1
Final Volume	10	10

Each sample was assessed in triplicate. Conditions for amplification were: 10 min at 95ºC, 40 cycles of 10 s at 95ºC and 7 s at 56ºC. The assay ended with a melting-curve program: 15 s at 95ºC, 1 min at 70ºC, then ramping to 95ºC while continuously monitoring fluorescence, and final cooling to 4ºC. Each pair of tumor and normal tissue was analyzed in the same QUAlu assay to minimize technical variation. Furthermore, each QUAlu assay was performed including a negative control without template and two positive controls consisting of two different starting amounts of genomic HCT116 DNA (10 ng and 5 ng). For each primer pair (qAlu and qL1PA) the efficiency value (E) per individual well was calculated by using the amplification curve kinetics (cpD2 method) [1]. The efficiency average for each pair of primers in every qPCR plate was used to obtain the percentage of unmethylated Alu elements (PUMA).

Determination of Alu elements containing the AACCCGG motif

First, data were downloaded from the UCSC MySQL database. Namely, Alu elements were retrieved as those elements from the RepeatMasker table (rmsk) [2] with repFamily "Alu" and repName starting by "Alu", to avoid FLAM and FRAM elements. Second, coordinates were transformed into sequence using the bedtools getfasta program [3] using the hg19 reference genome assembly. Third, sequences were scanned for the motif with the EMBOSS program fuzznuc [4] without allowing neither mismatches nor gaps. The search covered both the direct and complement strands. We reported the Alu elements containing the AACCCGG pattern, not the individual hits. The run counted 173 792 hits on 172 574 different Alu elements, as some Alu elements have more than a single copy of the motif.

Adaptor and primers design

The complementary oligonucleotides that made up the adaptor (ADP1 5'- AAAGCTCTGA -3' and the 5' phosphorylated ADP2 5'-CGTCAGAGCTTTGCGAAT -3' (Invitrogen) were designed to generate a cohesive end with a 5' CG overhang compatible with the 3' GC overhang produced by the *Hpa*II/*Msp*I digestion (Supplementary Fig. S1). The synthetic adaptor was prepared by incubating two complementary oligonucleotides at 65ºC for 2 minutes, and then cooling to room temperature for 35 minutes.

qAlu primers were specifically designed to amplify Alu elements. The reverse primer (qAlu Rv: 5'-ATTCGCAAAGCTCTGACGGGTT -3') was homologous to the synthetic adaptor, but additionally it was extended with the 5'- GGGTT -3' sequence complementary to the consensus Alu sequence 5'-AACCC -3' (Supplementary Fig. S1). The forward primer was devised to anneal within an Alu consensus sequence located upstream of the *Hpa*II/*Msp*I site. By bioinformatic analysis of the HG19 human genome assembly, we found 172 574 Alu repeats perfectly fulfilling QUAlu sequence features (presence of the 5'-AACCCGG -3' sequence containing the CCGG restriction site, see previous section).

To evaluate the adequacy of the design we performed a virtual experiment by digesting the 172 574 Alu repeats with *Msp*I (C\CGG) and subsequent concatenation to the ADP2 adaptor at the 3' end. One thousand random sequences of this subset were aligned with ClustalW using the default parameters. Then different Alu forward primers were designed and used together with the reverse primer (qAlu Rv) to perform an electronic PCR on the different concatenated Alu fragments [5], allowing 3 mismatches on the Alu forward primer and limiting the generated products from 50 to 200 bp. The selected forward primer (qAlu Fw: 5'-AGCTACTCGGGAGGCTGAG -3') matched 155 878 amplicons of 58 bp. The set of scripts used to perform this analysis is available at the qualu sourceforge repository (http://sourceforge.net/projects/qalu/).

The primers used in qL1PA PCR were designed by Terribas et al. [6].

Ion Torrent library preparation, sequencing and analysis

Ion Torrent specific sequences A and P1 (Supplementary Table S3) were fused to QUAlu amplicons following manufacturer's procedures (Ion Amplicon Library Preparation (Fusion Method), Life Technologies). Briefly, 1 uL of qAlu H amplicons and 1 uL of qAlu M amplicons of each sample were used to perform 20 cycles of quantitative PCR (LightCycler® Real-Time PCR System, Roche) with qAlu H and qAlu M primers, as follows: 5 uL of LightCycler® 480 SYBR Green I Master (Roche), 1 umol/L of forward primer and 1 umol/L of reverse primer, in a final volume of 10 uL. Then 1 uL of this product was used to perform 15 additional cycles in the same conditions but using Ion Torrent fusion primers (Supplementary Table S2).

Amplicons were purified (High Pure PCR Product Purification Kit, Roche), analyzed by Bioanalyzer (DNA 1000 assay, Agilent), and the peaks around the expected size were pooled for multiplexed sequencing. Next, Ion Sphere™ Particles (ISPs) were prepared accordingly to the Ion PGM™ Template OT2 200 Kit protocol (Life Technologies) using the Ion OneTouch™ 2 System (Life Technologies). Finally, ISPs were sequenced using the Personal Genome Machine® (PGM) System (Ion Torrent, Life Technologies) following the manufacturer's procedures (Ion PGM Hi-Q Sequencing Kit, Life Technologies). Ion Torrent primer sequences (A and P1) were removed from the reads using *cutadapt* (https://code.google.com/p/cutadapt/).

Effect of DNA degradation on QUAlu performance

HCT116 high quality DNA and aliquots sheared by enzymatic digestion with *Mse*I (NewEngland Biolabs)(2 ug of genomic DNA digested at 37ºC overnight) or by sonication (UCD300 Bioruptor Next Generation System (Diagenode, Seraing, Belgium); 25 and 45 cycles of sonication, 60 sec on / 60 sec off) were analyzed by QUAlu using standard conditions (see above).

Standard curve and relative sensitivity of QUAlu versus a single copy locus

Serial dilutions of HCT116 genomic DNA ranging from 25 ng to 0.005 pg of starting material per PCR tube were analyzed by qAlu M to determine the relative performance of this qPCR against a single copy locus (specifically the promoter region of Digestive organ expansion factor homolog (zebrafish) –*DIEXF*- gene, HGNC: 28440; accession number NM_0143388). The assay was performed in a LightCycler® 480 Real-Time PCR System with white Multiwell Plate 384 plates (Roche) in a final volume of 10 uL as follows: 5 uL of LightCycler® 480 SYBR Green I Master (Roche), 1 umol/L of *DIEXF* forward and reverse primers (5'-AACACTAGTGAACGAACT -3' and 5'- AGGAAGTTGTCCAGTCAA -3') and 1 uL of template. DIEXF amplicon length was 181 bp. The qPCR efficiencies per individual well, calculated as previously described, were 2.1 for qAlu M and 1.8 for *DIEXF*.

Tips and troubleshooting

- Input DNA: for optimal results, the amount of input DNA should be between 0.3 and 80 ng/uL. If input DNA concentration is too low to be quantified or is unknown you can proceed with 1uL of DNA.
- Adaptor preparation: Incubate 5 uL of ADP1 (100 nmol/L) and 5 uL of ADP2 (100 nmol/L) at 65ºC for 2 minutes and then cool to room temperature for 35 min. Add 990 uL of nuclease-free water and mix thoroughly to obtain 1mL of adaptor (1 nmol/L). Prepare single-use aliquots and store at -20 ºC.
- 10X Tango buffer preparation: thaw commercial buffer following manufacturer's instructions, prepare single-use aliquots and store at -20ºC.
- 10X T4 ligase buffer preparation: thaw commercial buffer following manufacturer's instruction, prepare single-use aliquots and store at -20ºC. Do not defrost 10X T4 ligase buffer aliquots more than twice.
- For best results, use freshly made digestion-ligation in the qPCR. Do not freeze the digestion-ligation.
- Always keep the tubes on ice.

- QUAlu is a very sensitive technique, thus always include a negative control (1uL of nuclease-free water instead of DNA) to detect possible contaminations. Negative controls should not amplify before cycle 34. Dismiss samples with quantification cycles similar to the negative control.
- Typical melting curves are shown below and samples with abnormal profiles should be discarded.

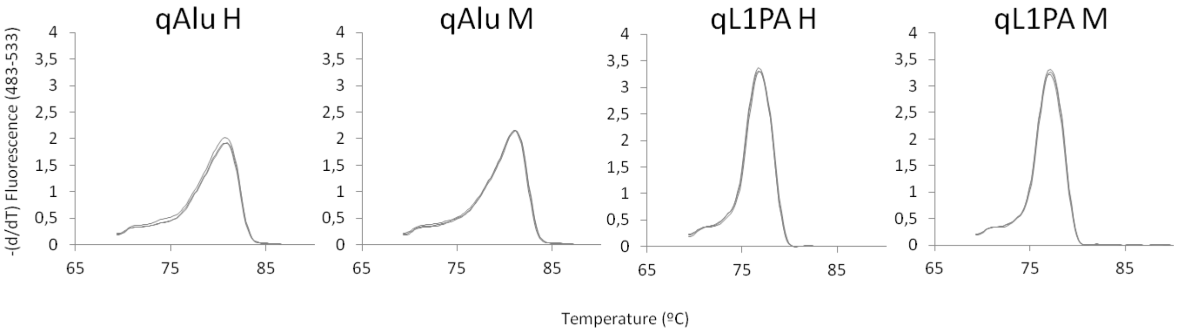

- Although QUAlu works in a wide range of concentrations, accurate comparisons should be performed with samples at similar DNA concentrations. When two samples are in very different ranges, it is recommended to repeat the quantification diluting the most concentrated sample.
- All the qPCRs related to a sample (qAlu for HpaII and for MspI and qL1PA for HpaII and for Msp I) should be analyzed in the same plate. All quantitative PCRs should be performed in triplicate.

List of abbreviations

Quantification of Unmethylated Alu (QUAlu)
qPCR using Alu primers of *Msp*I digested DNA (qAlu M)
qPCR using Alu primers of *Hpa*II digested DNA (qAlu H)
qPCR using L1PA primers of *Msp*I digested DNA (qL1PA M)
qPCR using L1PA primers of *Hpa*II digested DNA (qL1PA H)
Percentage of UnMethylated Alu elements (PUMA)
Quantification Cycle (Cq)

(a)

Alu consensus sequence:

```
GGCCGGGCGCGGTGGCTCACGCCTGTAATCCCAGCACTTTGGGAGGCCGAGGCGGGCGGATCACCTGAGGTCAGGAGTTCGAG
ACCAGCCGGCCAACACGGTGAAACCCGTCTCTACTAAAAATACAAAAATTAGCGGGCGTGGTGGCGCGCGCCTGTAATCCCAG
CTACTCGGGAGGCTGAGGCAGGAGAATCGCTTGAACCCGGGAGGCGGAGGTTGCAGTGAGCCGAGATCGCGCCACTGCACTCC
AGCCTGGGCGACAGAGCGAGACTCCGTCTC
```

(b)

QUAlu assay:

MspI/HpaII site*:* **C/CGG**
Adaptor 1 (ADP1): 5’- AAAGCTCTGA -3’
Adaptor 2 (ADP2): 5’- CGTCAGAGCTTTGCGAAT -3’
qAlu Fw: 5’- AGCTACTCGGGAGGCTGAG -3’
qAlu Rv: 5’- ATTCGCAAAGCTCTGACGGGTT -3’

Genomic DNA around the *Msp*I/*Hpa*II site:

```
5’- CGCCTGTAATCCCAGCTACTCGGGAGGCTGAGGCAGGAGAATCGCTTGAACCCGGGAGGCGGAG -3’
3’- GCGGACATTAGGGTCGATGAGCCCTCCGACTCCGTCCTCTTAGCGAACTTGGGCCCTCCGCCTC -3’
```

DNA fragment digested with *Msp*I or *Hpa*II:

```
5’- CGCCTGTAATCCCAGCTACTCGGGAGGCTGAGGCAGGAGAATCGCTTGAACC   -3’
3’- GCGGACATTAGGGTCGATGAGCCCTCCGACTCCGTCCTCTTAGCGAACTTGGGC -5’
```

Adaptor preparation:

```
5’- CGTCAGAGCTTTGCGAAT -3’
       ::::::::::
3’-    AGTCTCGAAA        -5’
```

***MspI* or *HpaII* digested DNA ligated to the adaptor:**

```
5’- CGCCTGTAATCCCAGCTACTCGGGAGGCTGAGGCAGGAGAATCGCTTGAACCCGTCAGAGCTTTGCGAAT -3’
3’- GCGGACATTAGGGTCGATGAGCCCTCCGACTCCGTCCTCTTAGCGAACTTGGGCAGTCTCGAAA        -5’
```

qPCR using Alu consensus primers:

```
                                                        3 -TTGGGCAGTCTCGAAACGCTTA -5’
5’- CGCCTGTAATCCCAGCTACTCGGGAGGCTGAGGCAGGAGAATCGCTTGAACCCGTCAGAGCTTTGCGAAT -3’
3’- GCGGACATTAGGGTCGATGAGCCCTCCGACTCCGTCCTCTTAGCGAACTTGGGCAGTCTCGAAA        -5’
             5’- AGCTACTCGGGAGGCTGAG -3´
```

Supplementary Figure S1. QUAlu digestion and ligation process. (A) The Alu consensus sequence was obtained from Weisenberger et al. [7]. (B) Scheme of the QUAlu assay with the detailed sequence of adaptor and primers. The sequence of the resulting amplicon is underlined.

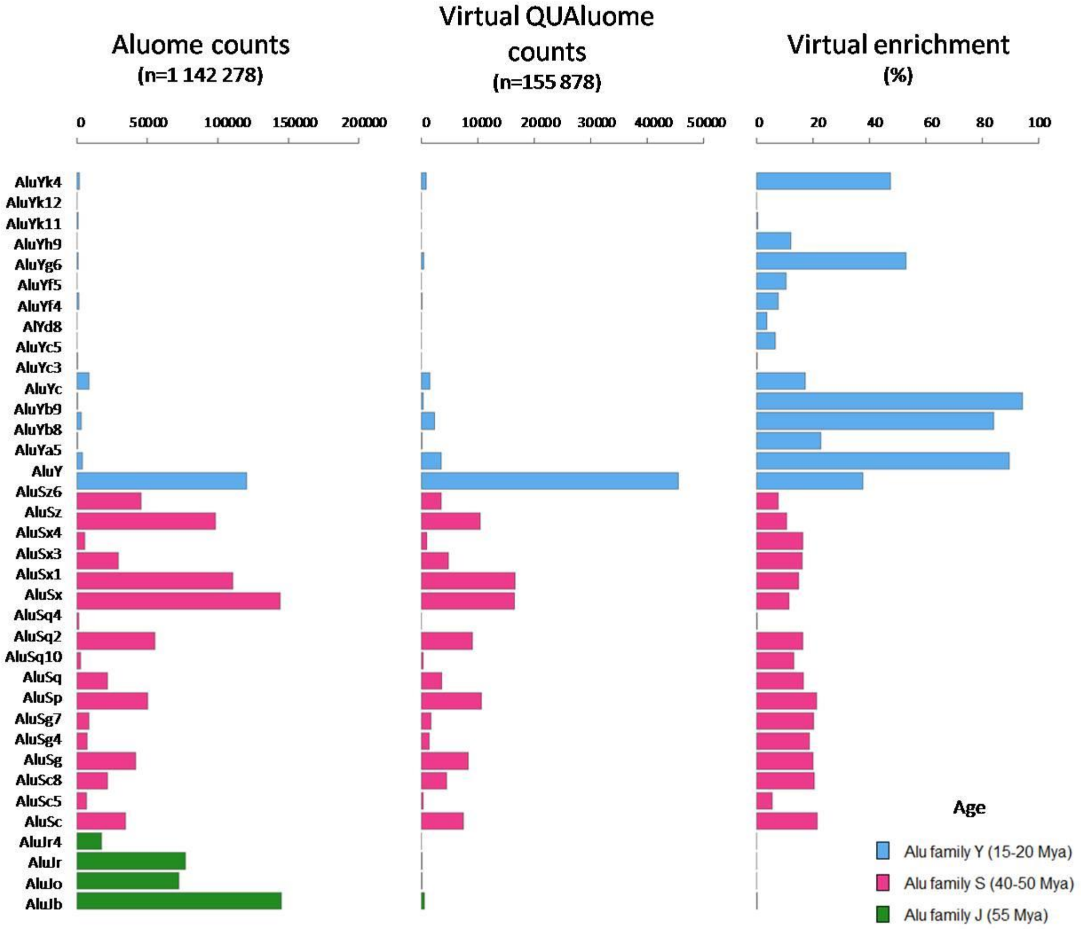

Supplementary Figure S2. Distribution of Alu elements per subfamilies in the different experimental subsets. Absolute number of Alu elements within the (A) human complete Aluome and (B) the virtual QUAluome. (C) QUAluome enrichment in comparison to Aluome. Subfamilies classification is according to RepeatMasker [2]. Alu families are sorted from younger to older and subfamilies by alphabetical order. Mya, million years ago.

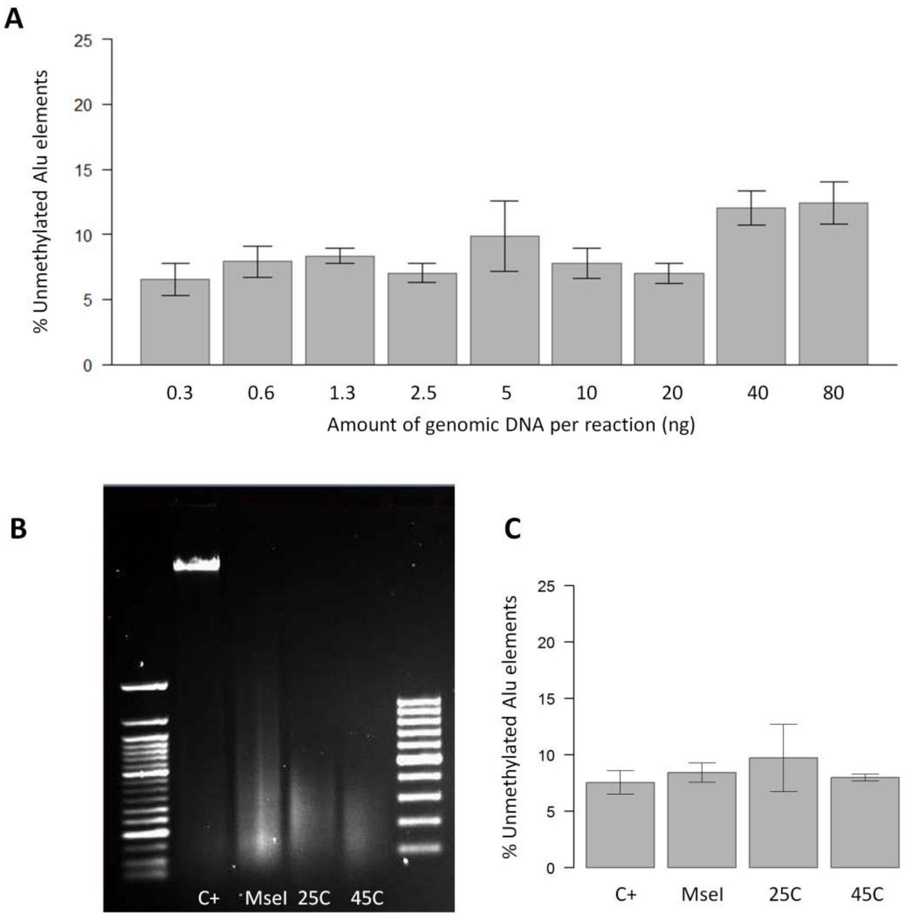

Supplementary Figure S3. QUAlu performance: linearity of response and influence of DNA integrity. (A) Quantification of the percentage of unmethylated Alu elements in the different points of the standard curve used in the linearity assessment. (B) Agarose gel (1%) showing different profiles of DNA degradation: non-degraded (C+), digested with *Mse*I, sonicated for 25 (*25C*) or sonicated for 45 cycles (*45C*). (C) Percentage of unmethylated Alu elements assessed by QUAlu in non degraded (C+) and degraded DNA.

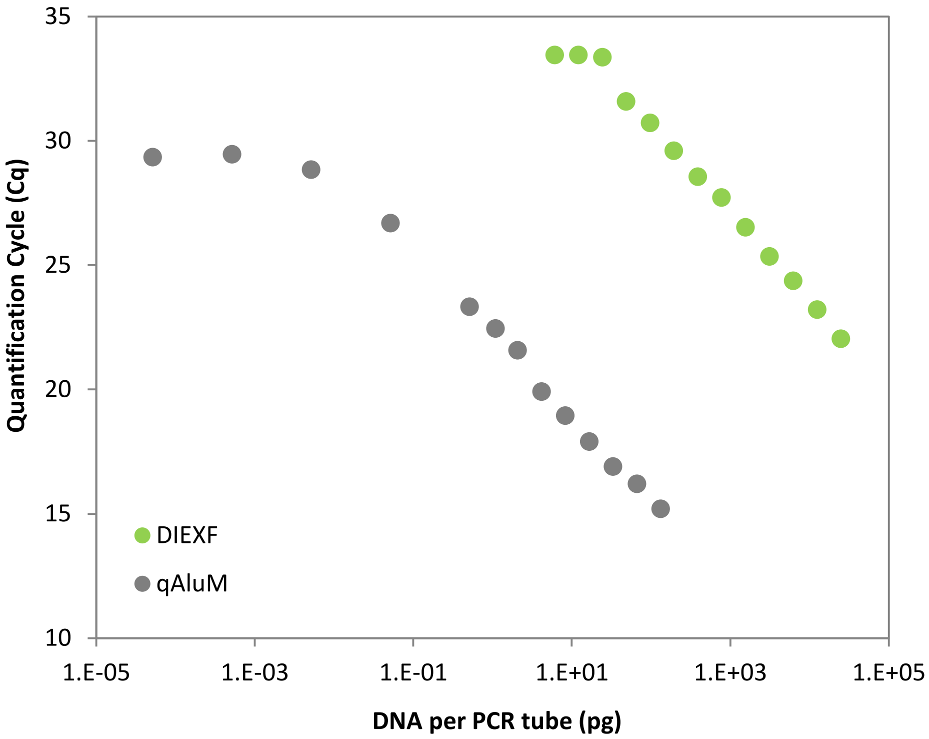

Supplementary Figure S4. qAlu M (gray) and a single copy locus (promoter region of DIEXF gene) (green) of serial dilutions of genomic DNA. qAlu amplification showed a linear response with dilutions of up to 0.005 pg of HCT116 genomic DNA per tube. DIEXF single locus amplification was linear up to 24 pg of HCT116 genomic DNA per tube.

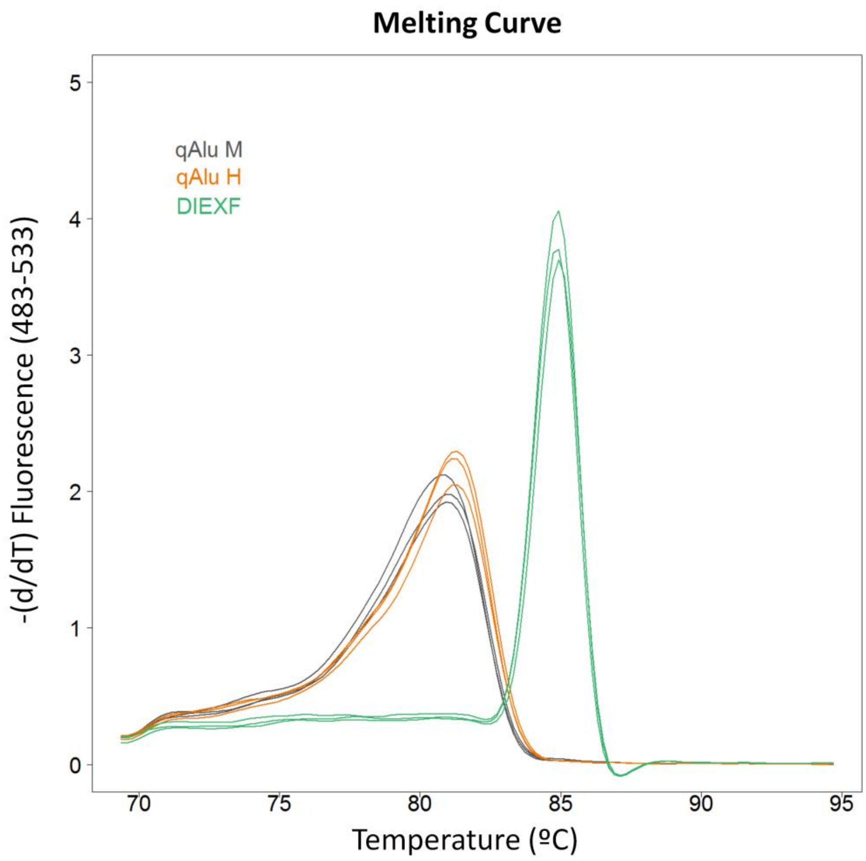

Supplementary Figure S5. Fluorescence melting peaks of qAlu M, qALu H and *DIEXF* qPCR in HCT116 genomic DNA. *qAlu M*: qPCR using the DNA obtained in MspI digested DNA in QUAlu assay. *qAlu H*: qPCR using the DNA obtained in HpaII digested DNA in QUAlu assay.

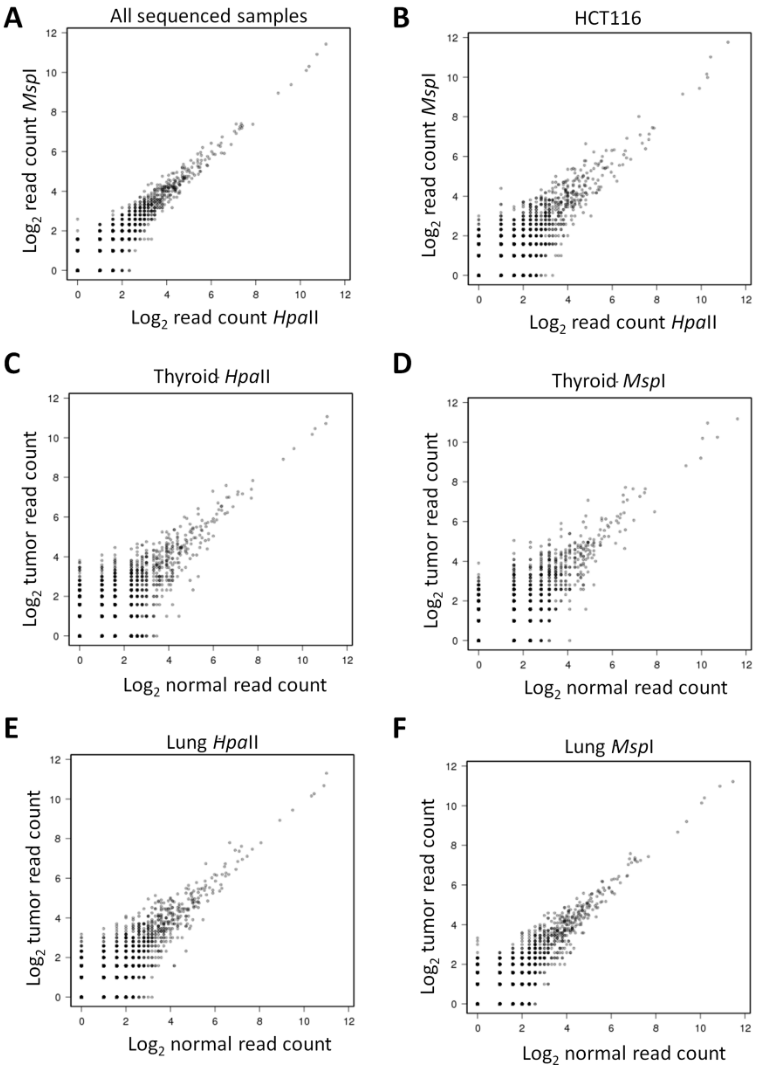

Supplementary Figure S6. Comparison of Next Generation Sequencing reads counts for QUAlu product. Each dot corresponds to a unique sequence. (A) Comparison between *Hpa*II and *Msp*I digestions data for all samples and (B) for HCT116 cell line. (C-F) Comparison between normal and tumor for thyroid and lung cancer samples digested with *HpaII* and *Msp*I. Reads count for each sample and each digestion condition were normalized by the sum of all reads.

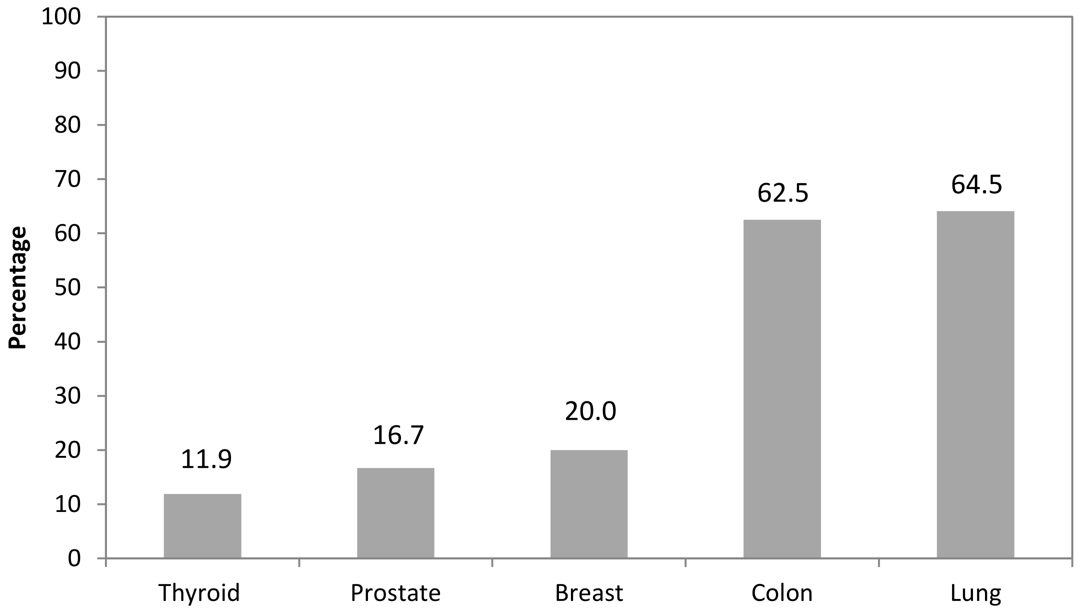

Supplementary Figure S7. Hypomethylated tumor samples. Fraction of hypomethylated tumor samples (PUMA>12) in five cancer types.

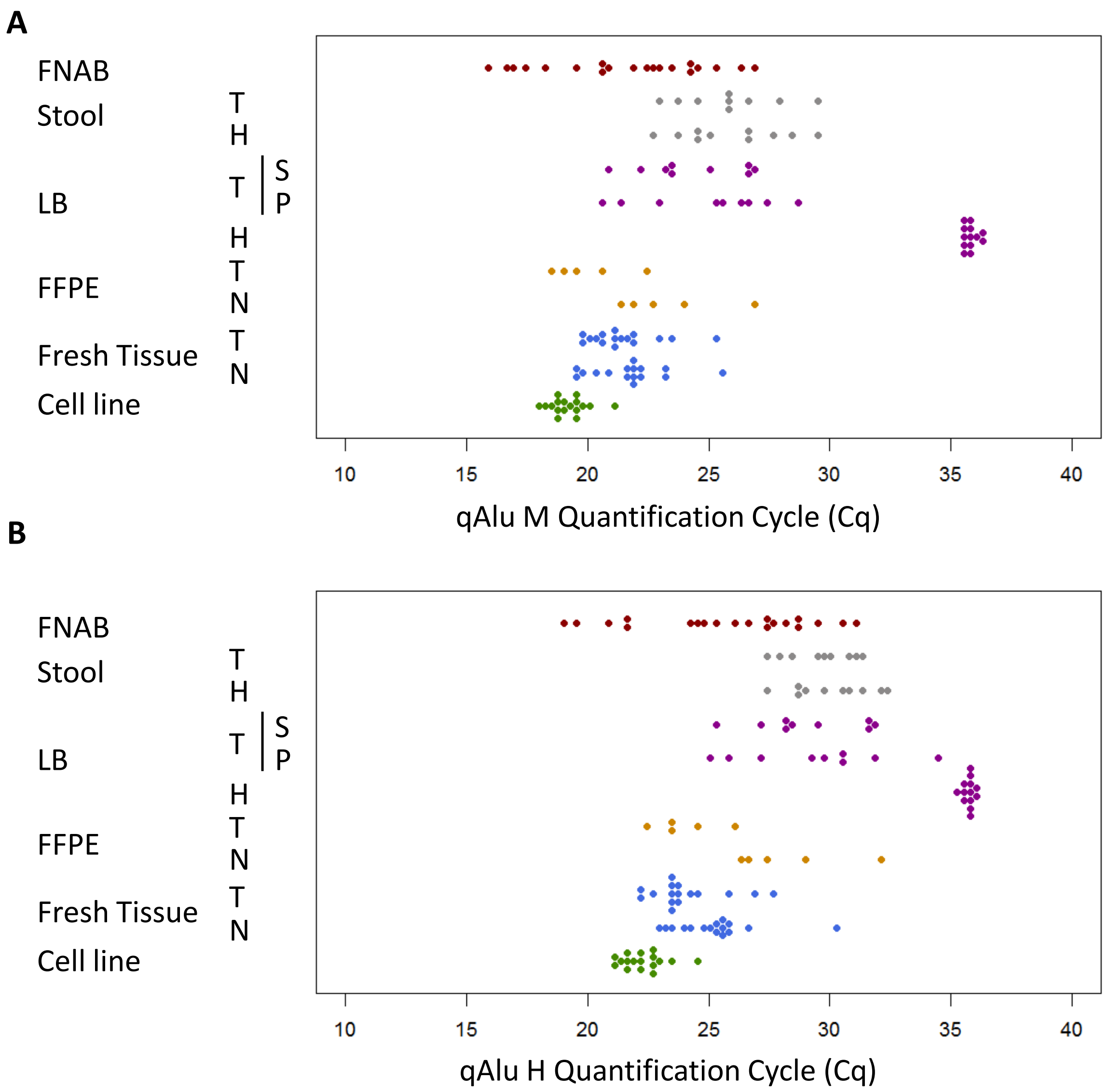

Supplementary Figure S8. Quantification cycles comparison among different sample types. (A) qAlu M and (B) qAlu H quantification cycles from: the colorectal carcinoma cell line HCT116 (Cell Line; considered the reference for high quality genomic DNA); normal (N) and tumor (T) colon fresh tissue; normal (N) and tumor (T) colon FFPE samples; healthy donors plasma (H), lung cancer patients plasma (P) and serum (S) liquid biopsies (LB); healthy donors (H) and colon cancer patients (T) stools; and thyroid goiter FNABs (FNAB).

SUPPLEMENTARY REFERENCES

1. Guescini M, Sisti D, Rocchi MB, Stocchi L and Stocchi V. A new real-time PCR method to overcome significant quantitative inaccuracy due to slight amplification inhibition. BMC Bioinformatics. 2008; 9:326.
2. Smit A, Hubley R and Green P. RepeatMasker Open-3.0. 1996. Computer program.
3. Quinlan AR and Hall IM. BEDTools: a flexible suite of utilities for comparing genomic features. Bioinformatics. 2010; 26(6):841-842.
4. Rice P, Longden I and Bleasby A. EMBOSS: the European Molecular Biology Open Software Suite. Trends Genet. 2000; 16(6):276-277.
5. Slater GS and Birney E. Automated generation of heuristics for biological sequence comparison. BMC Bioinformatics. 2005; 6:31.
6. Terribas E, Garcia-Linares C, Lazaro C and Serra E. Probe-based quantitative PCR assay for detecting constitutional and somatic deletions in the NF1 gene: application to genetic testing and tumor analysis. Clin Chem. 2013; 59(6):928-937.
7. Weisenberger DJ, Campan M, Long TI, Kim M, Woods C, Fiala E, Ehrlich M and Laird PW. Analysis of repetitive element DNA methylation by MethyLight. Nucleic Acids Res. 2005; 33(21):6823-6836.
